# Extended Thromboprophylaxis in Hospitalized Patients with Heart Failure: A Post Hoc Analysis of the MAGELLAN Study

**DOI:** 10.1055/a-1926-2489

**Published:** 2022-10-07

**Authors:** Alex C. Spyropoulos, Gary E. Raskob, Theodore E. Spiro, Wentao Lu, Yoriko De Sanctis, John Albanese, Alexandre Mebazaa, Elliot S. Barnathan

**Affiliations:** 1 Department of Medicine, The Donald and Barbara Zucker School of Medicine at Hofstra/Northwell, The Feinstein Institute for Medical Research, Manhasset, New York, United States; 2 Department of Medicine, Anticoagulation and Clinical Thrombosis Services, Northwell Health at Lenox Hill Hospital, New York, New York, United States; 3 Hudson College of Public Health, The University of Oklahoma Health Sciences Center, Oklahoma City, Oklahoma, United States; 4 Clinical Development, Pharmaceuticals, Bayer US LLC, Whippany, New Jersey, United States; 5 Janssen Research & Development, LLC, Raritan, New Jersey, United States; 6 Department of Anesthesia and Critical Care, University of Paris, Paris, France

**Keywords:** heart failure, thrombosis, rivaroxaban, direct oral anticoagulants, venous thromboembolism, medically ill

## Abstract

This post hoc subgroup analysis examined efficacy and safety outcomes with extended thromboprophylaxis rivaroxaban compared with in-hospital enoxaparin in 2,078 patients from the MAGELLAN study who had a hospitalization for heart failure or a history of heart failure and a lower risk of bleeding. A significant 36% reduction in the composite endpoint of asymptomatic proximal deep vein thrombosis (DVT) in the lower extremity, symptomatic DVT in the lower extremity (proximal or distal), symptomatic nonfatal pulmonary embolism, and venous thromboembolism-related death was observed with rivaroxaban. Major bleeding was low in both groups and not significantly increased with rivaroxaban.

## Introduction

Heart failure (HF) is characterized by a prothrombotic state, which not only increases the risk for cardioembolic events and ischemic stroke, 1 but also increases the risk of venous thromboembolism (VTE). 2 In a meta-analysis of 26 studies, the stroke rate among patients with HF (18/1,000) was substantially higher than the general population (1.5/1,000) and higher than that of patients with atrial fibrillation receiving anticoagulation (14/1,000). 3 An analysis of medically ill patients found the highest rates of VTE in patients with cancer (7.6%) and HF (5.6%). 4 VTE is the leading preventable cause of death in hospitalized patients and comprises diagnoses of deep vein thrombosis (DVT) and pulmonary embolism (PE), with a lifetime risk of approximately 8%. 5 Numerous risk factors, including comorbidities and recent hospitalization and immobilization, may contribute to VTE development. 6 In hospitalized HF patients, those who have more severe HF, as defined by elevated D-dimers (1.7 μg/mL) and N-terminal pro-brain natriuretic peptide (NT-pro-BNP, ≥ 1,906 pg/mL) as biomarkers, also have an increased risk of VTE compared with patients who have less severe or no HF at day 10 (4.3% vs. 2.2%) and day 35 (7.2% vs. 4.1%). 7

While it is recommended that acutely ill medical patients, including those with HF, receive thromboprophylaxis during hospitalization, guidelines do not recommend extended postdischarge thromboprophylaxis despite an increased risk of VTE in the postdischarge period due to an uncertain net clinical benefit stemming from an excess bleeding risk. 8 9 Rivaroxaban at the 10 mg dose was approved in 2019 by the U.S. Food and Drug Administration for primary thromboprophylaxis begun in hospital and continued after discharge for a total of 31 to 39 days in acutely ill medical patients at risk for thromboembolic complications who are at lower risk of bleeding. 10

The MAGELLAN trial was a multicenter, randomized, double-blind, parallel-group study of extended thromboprophylaxis in hospitalized medical patients with rivaroxaban (10 mg once daily) for 35 ± 4 days versus enoxaparin (40 mg once daily) for 10 ± 4 days. 11 Eligible patients had risk factors for VTE, including a history of HF. Results demonstrated that extended treatment with rivaroxaban was noninferior to enoxaparin for reducing the risk of VTE in acutely ill medical patients in the hospital at day 10 and superior at day 35, including in those with HF, but with excess major and nonmajor clinically relevant bleeding (NMCRB). 11 A retrospective analysis of the MAGELLAN study identified five key exclusion criteria that could be used to identify a subpopulation with reduced bleeding; these exclusion criteria were active cancer, bronchiectasis, active gastrointestinal ulcer, history of bleeding within 3 months, or the use of dual antiplatelet therapy. 12 13

The role of extended thromboprophylaxis in patients with HF in the posthospital discharge period is not well defined. This post hoc analysis sought to investigate the efficacy and safety of rivaroxaban in a subgroup of patients with HF from the MAGELLAN study that was previously identified as having a lower risk of bleeding.

## Materials and Methods

### Study Design

The study design and methods of the MAGELLAN study (ClinicalTrials.gov identifier: NCT00571649) have been reported previously. 11 Briefly, the MAGELLAN study evaluated the efficacy and safety of rivaroxaban (10 mg once daily) administered for 35 ± 4 days to enoxaparin (40 mg once daily) for 10 ± 4 days followed by placebo. Eligible patients were adults aged ≥ 40 years who were at risk of VTE due to moderate or severe immobility and had additional risk factors for VTE, such as prolonged immobilization, hospitalization for an acute medical illness, age ≥ 75 years, history of cancer, history of VTE, history of HF, thrombophilia, acute infectious disease contributing to the hospitalization, and body mass index ≥ 35 kg/m ^2^ . The original clinical trial was conducted in accordance with the Declaration of Helsinki and local regulations, with its protocol approved by the relevant local institutional review boards and ethics committees, and written informed consent was obtained from each patient before any study-specific procedures were performed.

This post hoc analysis identified a subgroup of patients who had a hospitalization for HF (New York Heart Association [NYHA] class III or IV) or an identified history of HF (NYHA class III or IV) and were at a lower risk of bleeding (MAGELLAN subpopulation safety, Supplementary Fig. S1 [online only]; that is, those who did not meet one of the five previously identified exclusion criteria). 13

### Study Outcomes

Efficacy outcomes were consistent with the overall study and included total VTE, which comprised the composite of asymptomatic proximal DVT in the lower extremity detected by mandatory bilateral lower extremity venous ultrasonography, symptomatic DVT in the lower extremity (proximal or distal), symptomatic nonfatal PE, and VTE-related death. Each efficacy component was also analyzed separately.

Safety outcomes included major bleeding, its components, and NMCRB events. Major bleeding defined by the International Society of Thrombosis and Haemostasis (ISTH) criteria was assessed in the safety population consisting of the on-treatment period plus 2 days. Efficacy and safety events were assessed by independent adjudication committees.

Risk differences for efficacy and safety outcomes, as well as numbers needed to treat (NNTs) and numbers needed to harm (NNHs), were calculated.

### Statistical Analyses

Baseline characteristics were summarized according to the modified intent-to-treat (mITT) day 35 population, defined as all randomized patients who received at least one dose of the study medication (safety population) and had an adequate assessment of VTE, including ultrasonography assessment at day 35. Efficacy analyses were conducted in the mITT day 35 population, and safety analyses were conducted in the safety population.

Relative risk ratios (RRs), including their corresponding confidence intervals (CIs), were calculated with geographic region stratification factor using the Mantel–Haenszel method for efficacy analyses. All analyses were done using SAS 9.4 Statistical Software.

## Results

### Patients

Of the 8,101 patients randomized, 7,998 were in the safety population in the MAGELLAN study, 1,551 (∼20%) were previously identified as having a higher risk of bleeding that included at least one of the five key bleed risk factors previously described. 12 13 The remaining 6,447 patients were at a lower risk of bleeding and comprised the MAGELLAN subpopulation. The subset of this subpopulation with HF included 2,078 patients of whom 1,017 were randomized to rivaroxaban and 1,061 were randomized to enoxaparin/placebo ( Supplementary Fig. S1 , online only). Baseline characteristics were generally similar between the rivaroxaban and enoxaparin/placebo groups in the HF subgroup ( Supplementary Table S1 , online only).

### Efficacy Outcomes

**Fig. 1:**
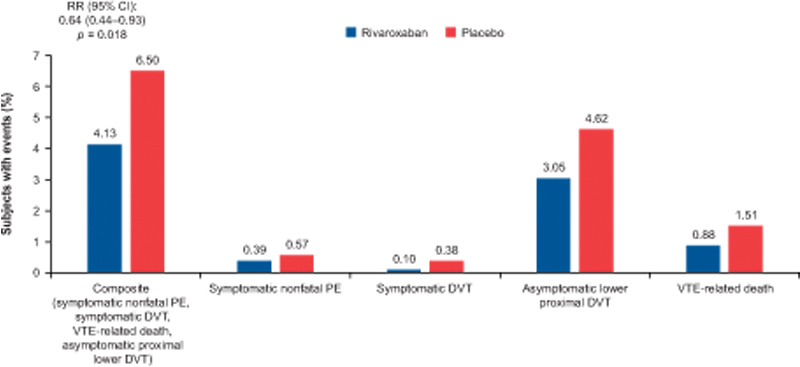
Relative risks of efficacy outcomes at mITT day 35. CI, confidence interval; DVT, deep venous thrombosis; mITT, modified intent-to-treat; PE, pulmonary embolism; RR, risk ratio; VTE, venous thromboembolism.

In the HF subgroup of the MAGELLAN subpopulation with a low risk of bleeding, the incidence of the composite efficacy endpoint at day 35 was significantly lower in the rivaroxaban group than in the enoxaparin/placebo group (4.13% vs. 6.50%; RR, 0.64; 95% CI, 0.44–0.93; *p* = 0.018; Fig. 1 ). All components of the primary efficacy endpoint numerically favored rivaroxaban. The predominant event in the rivaroxaban and enoxaparin/placebo groups was asymptomatic lower extremity proximal DVT (3.05 and 4.62%, respectively). The rivaroxaban group had a numerically lower incidence of VTE-related death compared with the enoxaparin/placebo group (0.88% vs. 1.51%).

### Safety Outcomes

**Fig. 2:**
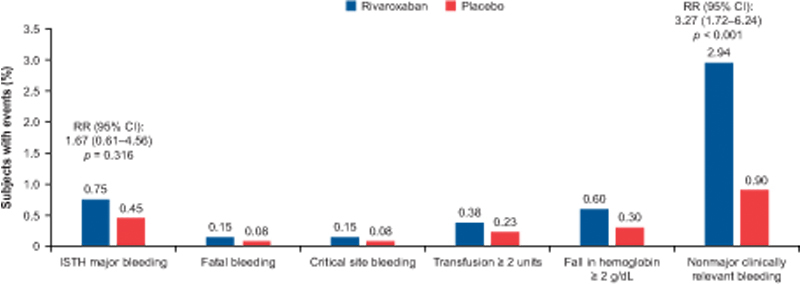
Relative risks of safety outcomes at mITT day 35. CI, confidence interval; ISTH, International Society of Thrombosis and Haemostasias; RR, risk ratio.

The risk of ISTH-defined major bleeding was low in both groups and not significantly increased with rivaroxaban compared with enoxaparin/placebo (0.75% vs. 0.45%; RR, 1.67; 95% CI, 0.61–4.56; *p* = 0.316; Fig. 2 ). ISTH-defined major bleeding was comprised mostly of transfusion ≥ 2 units and/or a fall in hemoglobin ≥ 2 g/dL. Fatal bleeding was low and comparable between groups. The risk of NMCRB was significantly higher in the rivaroxaban group as compared with the enoxaparin/placebo group (2.94% vs. 0.90%; RR, 3.27; 95% CI, 1.72–6.24; *p* < 0.001).

### Benefit–Risk Profile

**Fig. 3:**
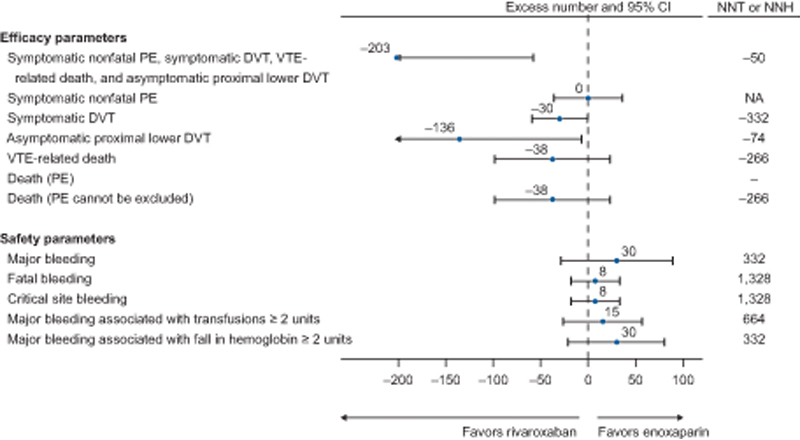
Risk differences based on proportion for 10,000 patients. CI, confidence interval; DVT, deep venous thrombosis; PE, pulmonary embolism; NA, not applicable; NNH, number needed to harm; NNT, number needed to treat; VTE, venous thromboembolism.

The benefit–risk profile was favorable, with an NNT to prevent one event in the composite endpoint of 50, while the NNH to cause one additional major bleed was 332 ( Fig. 3 ).

## Discussion

This post hoc analysis assessed the efficacy and safety of rivaroxaban in a subgroup of patients hospitalized with HF from the MAGELLAN study. This analysis demonstrated that, among patients hospitalized with HF and selected according to their low bleeding risk profile, extended thromboprophylaxis with rivaroxaban reduced the occurrence of VTE without an increased risk of major bleeding compared with standard duration in-hospital enoxaparin. There was a significant benefit seen with rivaroxaban compared with enoxaparin for the composite efficacy endpoint of asymptomatic proximal and symptomatic lower extremity DVT, symptomatic nonfatal PE, and VTE-related death. There was no increased risk of ISTH-defined major bleeding, including fatal bleeding, although there was an increased risk of NMCRB in the rivaroxaban group. The benefit–risk profile was favorable, with rivaroxaban showing an estimated NNT of 50 and an estimated NNH of 332.

These results are consistent with those seen in the overall MAGELLAN study, which showed superiority of rivaroxaban to enoxaparin/placebo for the primary efficacy composite endpoint (RR reduction = 23%; *p* = 0.0211) although bleeding was increased. 11 The risk of NMCRB in this study (2.9% vs. 0.9% with rivaroxaban vs. enoxaparin/placebo, respectively) was consistent with results from the overall MAGELLAN study (2.2% vs. 0.9%) and the MAGELLAN subpopulation with lower bleeding risk (2.1% vs. 0.7%). 13 Recently published data from the MAGELLAN and MARINER studies suggests that major bleeding, but not NMCRB, is consistently associated with risk of mortality in hospitalized medically ill patients. Hazard ratios (95% CI) for major bleeding versus no bleeding were 8.53 (5.61–12.97) for MAGELLAN and 3.46 (1.24–9.61) for MARINER, while results for NMCRB versus no bleeding were 1.74 (1.09–2.77) and 0.43 (0.10–1.74), respectively. 14 Thus, major bleeding remains the appropriate safety pairing to total VTE in an analysis of harms versus benefits of an extended thromboprophylactic strategy. 12

As demonstrated in a previous subgroup analysis of the MAGELLAN study, patients with more severe HF, as defined by high NT-pro-BNP, were at increased risk of VTE and derived benefit from rivaroxaban. 7 Findings from the current analysis in patients with HF selected on the basis of their lower bleeding risk further illustrate the importance of patient selection when using thromboprophylaxis for VTE events in medically ill patients with HF. Thus, HF patient profiles with more severe HF and lower risk of bleeding as defined by the current analysis remain ideal candidates that would derive net clinical benefit from extended posthospital discharge thromboprophylaxis with rivaroxaban.

As this is a post hoc study, results need to be interpreted within its limitations. The inclusion and exclusion criteria of MAGELLAN may limit generalizability to certain patient populations. For example, only patients ≥ 40 years of age who were hospitalized for an acute medical illness were enrolled. The identification of patients with HF and a lower risk of bleeding were based on criteria (e.g., hospitalization or identified history) and not prospectively assessed clinically. Given this, further prospective studies will be needed to corroborate these results.

In conclusion, in a carefully selected population of patients with HF at a lower risk of bleeding, there was an increased risk of VTE and VTE-related death that is reduced using extended thromboprophylaxis with rivaroxaban without a significant increased risk of major bleeding.
