# Supplementary figures and images for: Extended Thromboprophylaxis in Hospitalized Patients with Heart Failure: A Post Hoc Analysis of the MAGELLAN Study
Source: TH Open. 2022 Oct 7;6(4):e304–8. doi: 10.1055/a-1926-2489 (PMC9546607; doi:10.1055/a-1926-2489)

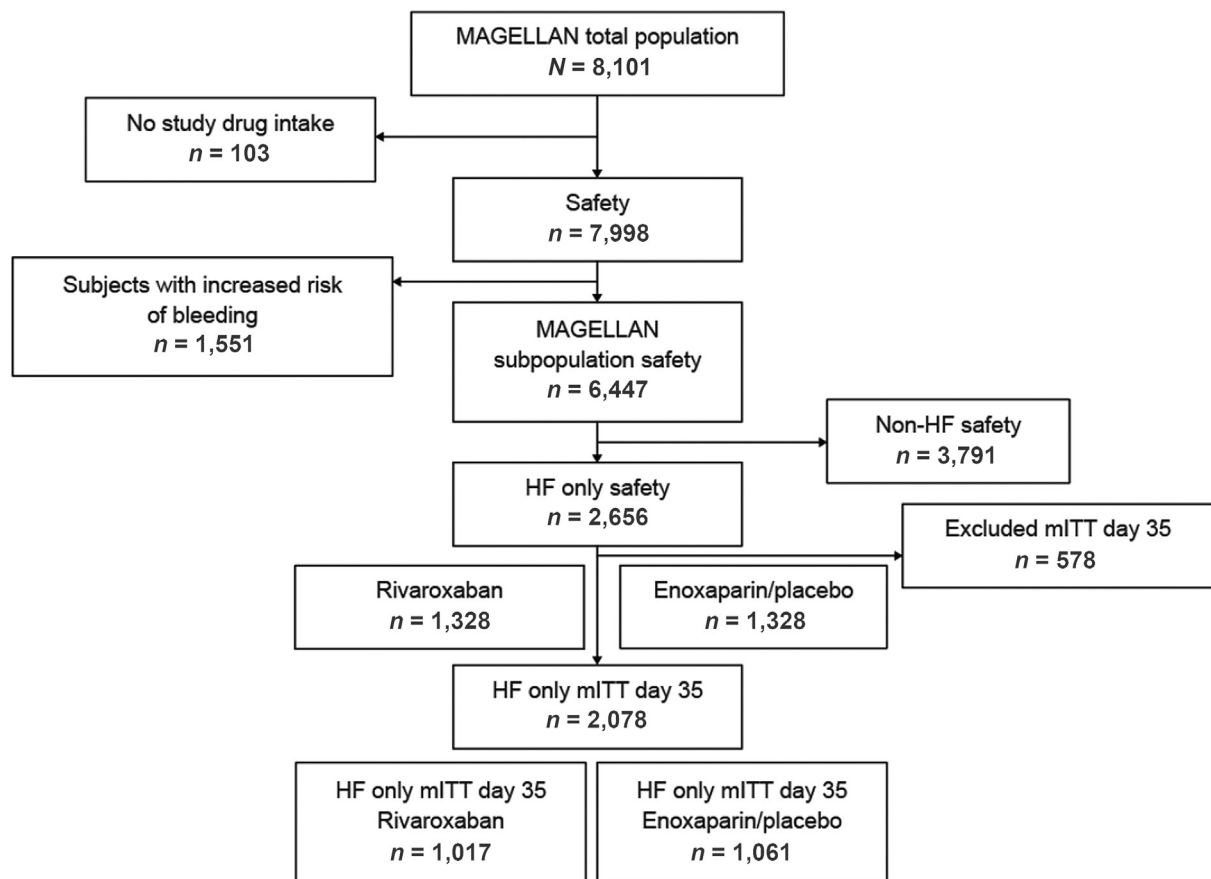

Supplementary Fig. S1 Patient disposition. D, day; HF, heart failure; mITT, modified intent-to-treat.

Supplement: Supplementary file 1 — Supplementary Material [file 10-1055-a-1926-2489-s22060027.pdf]
